# The Fish Pathogen *Vibrio ordalii* Under Iron Deprivation Produces the Siderophore Piscibactin

**DOI:** 10.3390/microorganisms7090313

**Published:** 2019-09-03

**Authors:** Pamela Ruiz, Miguel Balado, Juan Carlos Fuentes-Monteverde, Alicia E. Toranzo, Jaime Rodríguez, Carlos Jiménez, Ruben Avendaño-Herrera, Manuel L. Lemos

**Affiliations:** 1 Laboratorio de Patología de Organismos Acuáticos y Biotecnología Acuícola, Facultad de Ciencias de la Vida, Universidad Andrés Bello, 2531015 Viña del Mar, Chile; 2 Interdisciplinary Center for Aquaculture Research (INCAR), 2531015 Viña del Mar, Chile; 3 Departamento de Microbiología y Parasitología, CIBUS-Facultad de Biología and Instituto de Acuicultura, Universidade de Santiago de Compostela, 15782 Santiago de Compostela, Spain; 4 Centro de Investigacións Científicas Avanzadas (CICA), Departamento de Química, Facultade de Ciencias, Universidade da Coruña, 15071 A Coruña, Spain

**Keywords:** *Vibrio ordalii*, fish pathogens, iron uptake, siderophores, piscibactin, vanchrobactin

## Abstract

*Vibrio ordalii* is the causative agent of vibriosis, mainly in salmonid fishes, and its virulence mechanisms are still not completely understood. In previous works we demonstrated that *V. ordalii* possess several iron uptake mechanisms based on heme utilization and siderophore production. The aim of the present work was to confirm the production and utilization of piscibactin as a siderophore by *V. ordalii*. Using genetic analysis, identification by peptide mass fingerprinting (PMF) of iron-regulated membrane proteins and chemical identification by LC-HRMS, we were able to clearly demonstrate that *V. ordalii* produces piscibactin under iron limitation. The synthesis and transport of this siderophore is encoded by a chromosomal gene cluster homologous to another one described in *V. anguillarum*, which also encodes the synthesis of piscibactin. Using β-galactosidase assays we were able to show that two potential promoters regulated by iron control the transcription of this gene cluster in *V. ordalii*. Moreover, biosynthetic and transport proteins corresponding to piscibactin synthesis and uptake could be identified in membrane fractions of *V. ordalii* cells grown under iron limitation. The synthesis of piscibactin was previously reported in other fish pathogens like *Photobacterium damselae* subsp. *piscicida* and *V. anguillarum*, which highlights the importance of this siderophore as a key virulence factor in Vibrionaceae bacteria infecting poikilothermic animals.

## 1. Introduction

*Vibrio ordalii* is a γ-proteobacterium which causes vibriosis, a hemorrhagic septicemia, in several species of aquacultured fish, mainly salmonids [1]. Although vibriosis outbreaks due to *V. ordalii* have been reported around the globe, in the last 15 years they reached an important impact in Chile, where they cause significant economic losses in salmonids aquaculture [2,3]. Besides its genetic similarity to *V. anguillarum* [4,5], another important fish pathogen with worldwide distribution, many aspects of the virulence mechanisms of *V. ordalii* still remain unknown. While its pathogenicity is not correlated to erythrocytes hemagglutination capacity or biofilm formation in Atlantic salmon (*Salmo salar*), the hydrophobic properties of *V. ordalii* cells could play a role in virulence. Moreover, *V. ordalii* can evade the host immune system and can survive within Atlantic salmon mucus, which likely facilitates colonization [3,6]. However, many aspects of its ability to colonize and multiply within the fish hosts remain unclear.

For most bacteria iron uptake ability during the naturally iron-limited conditions of an infection is a key virulence factor essential for multiplication within the host [7,8,9]. Besides the importance of iron for the cell metabolism, this element is an important signal that regulates expression of many other metabolic and virulence functions in bacterial cells [10]. This regulation is usually mediated by the transcriptional regulator Fur which needs Fe^2+^ as cofactor to bind to the promoter region of genes controlled by iron levels and prevent the binding of RNA polymerase to DNA [11]. The main mechanisms described in Gram-negative bacteria to get iron from the cell surroundings are the direct use of heme groups as a source of iron [12] and the synthesis of siderophores, which can efficiently sequester the iron bound by transferrins and other iron-holding proteins within the host [9,13,14]. The ferri-siderophore is then internalized through specific TonB-dependent outer membrane protein receptors that are energized through the TonB system [15,16,17]. Bacterial fish pathogens are not an exception for iron requirements and several mechanisms of iron uptake, including the use of heme and the synthesis of siderophores, have been reported in many of these bacteria [18,19,20,21,22,23,24,25].

We have previously demonstrated that *V. ordalii* can also use heme and hemoglobin as iron sources and that it has the ability to produce siderophores [26]. However, despite the clear relationship between *V. ordalii* iron uptake ability and pathogenicity, the precise nature of the iron assimilation mechanisms remains unclear. In this previous work, from genetic and genomic analysis, the results of cross-feeding assays, and from some other data in the literature [4], we suggested that *V. ordalii* could likely produce piscibactin as a siderophore. Piscibactin was isolated and characterized from the fish pathogen *Photobacterium damselae* subsp. *piscicida* [23]. In this bacterium piscibactin synthesis is encoded in a pathogenicity island harbored in the pPHDP70 virulence plasmid [27]. Recent in silico genomic studies in the Vibrionaceae family showed that the gene cluster encoding piscibactin synthesis and transport is really widespread in many species of *Vibrio* and *Photobacterium* [28]. In fact, we have recently demonstrated that some strains of *V. anguillarum*, a bacterium closely related to *V. ordalii*, produces piscibactin in a temperature-dependent fashion, being preferentially expressed at low temperatures. In these conditions piscibactin synthesis is a key virulence factor for *V. anguillarum* [29].

In the present work, we have characterized the gene cluster encoding the biosynthesis and transport of piscibactin and demonstrated, by genetic, proteomic and chemical analysis, that piscibactin is indeed produced as siderophore by *V. ordalii*.

## 2. Materials and Methods 

### 2.1. Bacterial Strains and Growth Conditions

Three *V. ordalii* strains were used: The type strain ATCC 33509^T^ and two strains, Vo-LM-13 and Vo-LM-18, previously isolated from vibriosis outbreaks in Atlantic salmon cultured in Chile [3,6]. All were confirmed as *V. ordalii* according to the PCR protocol previously described [30]. All strains were routinely cultivated on Trypticase Soy Agar or Trypticase Soy Broth supplemented with 1% (w/v) NaCl (TSA-1 and TSB-1, respectively). For some experiments the CM9 minimal medium was also used [31]. Stock cultures were kept frozen at −80 °C in Criobilles tubes (AES Laboratories, Combourg, France) or in TSB-1 with 15% (v/v) glycerol.

### 2.2. RNA Extraction and RT-PCR

To analyze the transcriptional regulation of the gene cluster involved in the biosynthesis and transport of the siderophore piscibactin, a RT-PCR was performed with the primers listed in Table 1. For this assay, *V. ordalii* Vo-LM-18 was grown in iron-limited (TSB-1 plus 2,2′-dipyridyl), iron-excess (TSB-1 plus FeCl_3_ 10 µM) and standard conditions (TSB-1). Total RNA was prepared from cultures after 48 h post-incubation using TRIzol^®^ reagent (Ambion-ThermoFisher, Waltham, MS, USA) according to the manufacturer’s instructions. Each RNA sample was subjected to treatment with DNase I RNase free. To obtain the cDNA, 5 µg total RNA and reverse transcriptase enzyme M-MLV (Invitrogen-ThermoFisher, Waltham, MS, USA) was used following the manufacturer’s instructions for each reverse transcription reaction. The PCR reaction was prepared with the cDNA, 1 U of BioTaq DNA polymerase (Bioline, Memphis, TN, USA), 200 µM of each dNTP and 2 mM MgCl_2_, final concentration. Depending on the melting temperature (Tm) of each pair of primers, annealing temperatures ranged from 55 to 60 °C. Times of elongation were selected based on the expected size of amplification (1 min·kb^−1^). In all cases, the same reaction mixture, but without reverse transcriptase, was used as negative control, and chromosomal DNA of the Vo-LM-18 strain was used as positive control.

### 2.3. Construction of lacZ Transcriptional Fusions and β-Galactosidase Assays

The presence of potential gene promoters within the piscibactin gene cluster of *V. ordalii* was performed using BPROM tool [32]. Putative *Fur boxes* were detected by an in silico search of the GATAAT hexamer [33]. DNA fragments corresponding to *V. ordalii frpA* and *araC1* promoter regions (P1 and P2, respectively) were amplified by PCR using primers specified in Table 1. The amplified fragments included the region upstream of the start codon and the first nucleotides (ca. 50 bp) of *frpA* or *araC1* coding sequences. These putative promoter regions were fused to a promoterless *lacZ* gene and inserted into the low-copy-number reporter plasmid pHRP309 [34]. The resulting transcriptional fusion constructs, *P1::lacZ* and *P2::lacZ*, were mobilized from *Escherichia coli* β3914 into *V. ordalii* Vo-LM-18 by conjugation. Transformed ex-conjugants were selected on the basis on their resistance to gentamicin (pHRP309 marker). As a negative control, *V. ordalii* Vo-LM-18 with an empty pHRP309 was used. To determine whether potential promoters were regulated by iron, a total of four growth conditions were tested for each one of the transcriptional fusions: Cells grown in CM9, cells grown under iron excess (CM9 plus FeCl_3_ 20 µM) and two iron limiting conditions, CM9 plus 2,2′-dipyridyl 25 mM and CM9 plus 2,2′-dipyridyl 80 µM. All cultures were carried out with agitation at 100 rpm at 18 °C until an OD_600_~0.1 to record the β-galactosidase activity.

The transcriptional activity was determined by measuring the β-galactosidase activity of fusions *P1::lacZ* and *P2::lacZ* following the method described by Miller [35]. Volumes of 0.1 and 0.5 mL, respectively, were used. Both were brought to a final volume of 1 mL with buffer Z (Na_2_HPO_4_ 2H_2_O 60 mM; NaH_2_PO_4_·H_2_O 40 mM; KCl 10 mM; MgSO_4_ 7H_2_O 1 mM and β-mercaptoethanol 50 mM; pH 7.0). To this mixture 20 µL of chloroform and 10 µL of a solution of 0.1% SDS were added and the final solution was incubated at 37 °C for 5 min. The reaction was initiated by adding 0.2 mL of ortho-nitrophenyl-β-galactoside (ONPG; 4 mg·mL^−1^ in Z buffer). The reaction was stopped with 0.5 mL of 1 M Na_2_CO_3_ when a color change to yellow was generated. Finally, A_420_ was measured in a UV-VIS spectrophotometer (Hitachi U2000, Tokyo, Japan).

### 2.4. Analysis of Outer Membrane Proteins (OMP) Profile of V. ordalii

OMPs were obtained from *V. ordalii* strains ATCC 33509^T^, Vo-LM-18, and Vo-LM-13 grown under iron excess (TSB-1) and iron limitation (TSB-1 + 2,2′-dipyridyl, using a concentration half of the specific MIC for each strain). Each strain was cultured in 500 mL of TSB-1 or TSB-1 + 2,2′-dipyridyl at 18 °C for 48 h. After incubation, the media were centrifuged at 10,000 × *g* for 10 min at 4 °C. The cell pellets were resuspended in 3 mL of a solution containing 10 mM Tris-HCl (pH 8.0), 0.3% NaCl and 1% of a protease inhibitor cocktail (Sigma-Aldrich, St. Louis, MO, USA). The suspension was then sonicated three times with a Branson 250 Sonifier (60 W pulses for 30 s, 30 s intervals in ice). After 1–2 min of centrifugation to eliminate cell debris, supernatants were centrifuged at 17,000 × *g* for 60 min at 4 °C. The pellets obtained contained total cell membranes.

Outer membrane fractions were obtained as previously described [36,37]. Briefly, the total membrane pellets were resuspended in a solution containing 20 mM Tris-HCl (pH 8.0), 3% (*w*/*v*) sodium lauryl sarcosinate (Sigma-Aldrich, St. Louis, MO, USA) and 1% protease inhibitor cocktail (Sigma-Aldrich, St. Louis, MO, USA). The suspension was incubated at room temperature for 20 min to dissolve the inner membrane. Outer membranes were pelleted by 100,000 × *g* ultracentrifugation for 60 min at 4 °C and washed twice with distilled water. Protein concentration was determined using the BCA Assay Kit (Thermo Scientific, Waltham, MS, USA), and samples were kept at −20 °C until use.

Iron-regulated OMP (IROMP) profiles were compared for each *V. ordalii* strain between cells grown with or without iron limitation. Each extract (20 μg) was mixed with the SDS-PAGE sample buffer, heated at 95 °C for 5 min, and separated by SDS-PAGE with 7.5% (w/v) acrylamide in the resolving gel. Electrophoresis was performed in a Mini-PROTEAN 3 Cell (Bio-Rad, Portland, ME, USA) at 120 V for 120 min. Protein bands were stained with 0.05% Coomassie blue R (Sigma-Aldrich, St. Louis, MO, USA) for at least 1 h and destained for 2 h in 10% methanol and 10% acetic acid. The relative mobility of each protein was determined by comparison with standard protein markers (Precision Plus Protein Standards, Bio-Rad). Digital images were collected using a G:BOX Chemi XT4 Fluorescent and Chemiluminescent Imaging System (Syngene, Frederick, MD, USA) with GeneSys automatic control software and GeneTools analysis software (Syngene, Frederick, MD, USA). Three independent separations, from two different cultures, were performed for each strain and growth condition. Further analyses were done only to protein bands induced or increased in intensity under iron-limited conditions.

### 2.5. Protein Identification by Peptide Mass Fingerprinting (PMF)

Candidate iron-regulated bands from SDS-PAGE gels were identified by PMF, using MALDI-TOF (Matrix-Assisted Laser Desorption/Ionization Time-of-Flight) Mass Spectrometry analysis, as previously described [38]. In brief, protein bands of interest were manually excised and subjected to in-gel digestion with trypsin using the In-Gel DigestZp Kit (Millipore ES, Madrid, Spain), following the manufacturer’s protocol, to extract proteins prior to mass spectrometry analysis. Before digestion, the samples were reduced with dithiothreitol and alkylated with iodoacetamide. Proteins were identified by PMF with an Ultraflex III TOF/TOF (Bruker ES, Madrid, Spain). For negative identifications, due to mixed proteins in a single band, a liquid chromatography ion-trap mass-spectrometer system with an amaZon speed ETD (Bruker ES, Madrid, Spain) was used. The SwissProt and NCBInr protein databases were screened with Mascot v2.3 (Matrix Science). The identified peptides were then subjected to a BLASTP analysis using the NCBI (National Center for Biotechnology Information) database, to search for homologues.

### 2.6. Bioinformatics Tools

The DNA and protein sequences were analyzed using the NCBI databases through the BLAST algorithms. The protein families database (Pfam 31.0) of EMBL-EBI (European Bioinformatics Institute) was used to predict the protein domain organization [39]. The functional promoters were identified using the online database BPROM. The organization of putative domains in biosynthetic proteins were detected using the PKS/NRPS database (http://nrps.igs.umaryland.edu/).

### 2.7. Detection of Siderophore Piscibactin

Piscibactin was detected as previously described [23] with slight modifications as follows: 1 L of cell-free culture broth of strain Vo-LM-18 was concentrated under vacuum (39 °C) until 300 mL. Then, 150 mL were transferred to a flat-bottom flask provided with a magnetic stir bar and 750 µL of a solution of GaBr_3_ in H_2_O (12 mg/mL) were added dropwise over 5 min and gently stirred for another 10 min. This solution was stored at 4 °C during 24 h. An aliquot of the solution containing piscibactin-Ga(III) complex (75 mL) was submitted to Solid Phase Extraction (SPE) through an OASIS^®^ (Waters, Cerdanyola del Vallès, Spain) cartridge (35 cm^3^, 6 g) using an extraction vacuum manifold (0.2 bar) and eluted with 30 mL of the following mixtures of H_2_O and CH_3_CN: 1:0; 1:3; 1:1; 0:1. Fractions were dried out under reduced pressure and subjected to LC-HRMS analysis using an Atlantis dC18 (100 mm × 4.6 mm, 5µm) column (Waters) at a flow rate of 1 mL/min. Separation, with a sample injection volume of 20 µL, was achieved by a 35 min gradient from 10% to 100% of CH_3_CN in H_2_O, then a 5 min isocratic step of 100% CH_3_CN. LC-ESI(+)-HRMS analysis of the fraction eluted with the mixture H_2_O/CH_3_CN 1:1, named as L3, showed a peak at 12.06 min that displayed the characteristic isotopic cluster of piscibactin-Ga(III) complex at *m*/*z* 518.9928/521.9913.

Results are reported following the identification requirements for MS techniques SANTE/11945/2015. Since it was possible detect both ions at significant intensity, the difference between the calculated and the detected exact mass of piscibactin-Ga(III) complex in ppm (∆*m*/*z*) and the isotopic ratio abundance error (δ RIA) of M + 1/M could be obtained using the Formulas (1) and (2). The quality of the spectral information was achieved by narrowing the detection *m*/*z* range around the compound of interest of 350–600 dalton measured in a LTQ-Orbitrap, which is in agreement with the small values δ RIA found.

Formula (1)—SI: Mass accuracy:

(1) $$\Delta \frac{m}{z}=\;\left|\frac{m\;measured-m\;theoretical}{m\;theoretical}\times 10^{6}ppm\right|.$$

Formula (2)—SI: Isotopic ion abundance ratio error (*δ RIA*):

(2) $$\delta \;RIA\left(\%\right)=\left|100\times \frac{RIA_{exp}-RIA_{theo}}{RIA_{theo}}\right|.$$

Presence of the siderophore vanchrobactin in the same cell-free culture supernatants was also detected using the methodology previously described [19,29].

### 2.8. Statistical Analysis

Data from all assays were statistically analyzed using analysis of variance (ANOVA). Significant differences were established as *p* < 0.05.

## 3. Results

### 3.1. Characterization of the V. ordalii Gene Cluster Encoding a Piscibactin-Like Siderophore

**Figure 1:**
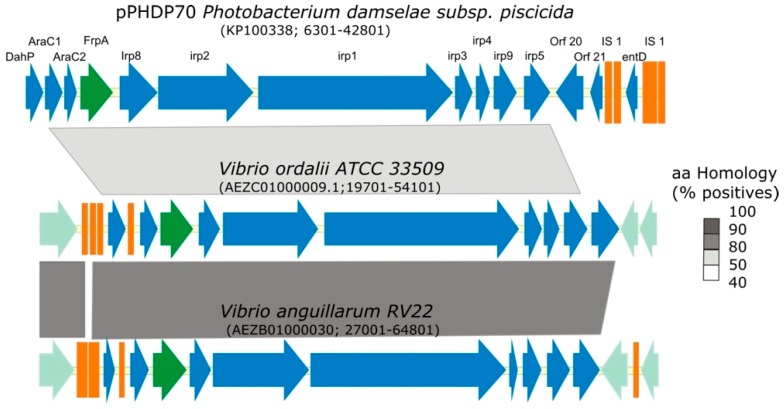
Comparative analysis of the *Vibrio ordalii* ATCC 33509^T^ *irp* gene cluster with the homologous chromosomal region of *V. anguillarum* RV22 and with the homologous sequence from plasmid pPHDP70 from *P. damselae* subsp. *piscicida*. Biosynthetic and regulatory genes are depicted in blue and the gene encoding the outer membrane receptor (FrpA) in green. Other genes and short ORFs are shown in clear green and orange colors. Grey blocks indicate percentages of similarity in the proteins sequence. The GenBank accession numbers and the nucleotide positions interval are indicated below the name of each species.

An in silico analysis of the genome of *V. ordalii* ATCC 33509 shows the presence of a gene cluster homologous to the piscibactin cluster (*irp_ang_*) described in the chromosome II of *V. anguillarum* RV22 [29]. Both clusters show a high degree of synteny and a similarity between 96% and 98% at the amino acid level (Figure 1). This genomic island includes the 11 genes (*irp* genes) previously identified as part of the plasmid encoding piscibactin in *P. damselae* subsp. *piscicida* [27] (Figure 1). An in silico search in GenBank, and previously published works [28], show the presence of homologous gene clusters in several members of the Vibrionaceae family, such as *V. cholerae*, *V. mimicus*, *V. coralliilyticus*, *V. anguillarum* or *Photobacterium profundum*. It is noteworthy that this gene cluster exhibits about a 40% similarity with the genes of the HPI pathogenicity island (encoding the synthesis of the siderophore yersiniabactin) of *Yersinia* spp. and it was reported as a key virulence factor for *P. damselae* subsp. *piscicida* [27,40].

**Figure 2:**
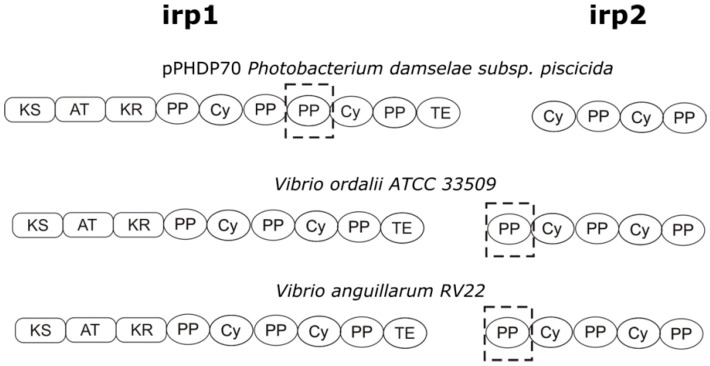
Representation of the catalytic domains predicted in Irp1 and Irp2 enzymes of *Photobacterium damselae* subsp. *piscicida*, *V. ordalii* ATCC 33509^T^ and *V. anguillarum* RV22. Analysis of domains was performed using the PKS/NRPS database (http://nrps.igs.umaryland.edu/). Abbreviations: AT, acyltransferase; Cy, cyclization; KS, ketoacil synthase; KR, ketoreductase; PP, peptidyl-carrier protein; TE, thioesterase. Dotted boxes highlight the main differences.

Piscibactin is synthetized by NRPS-type (non-ribosomal peptide synthetases) enzymes encoded by *irp1* and *irp2* genes [23]. The bioinformatic analysis of the resulting proteins Irp1 and Irp2 of *V. ordalii* showed that the catalytic domains present in these enzymes are almost identical, with a similarity in the amino acid sequence of 99%, to their counterparts encoded by *irp_ang_* cluster of *V. anguillarum* RV22 [29] (Figure 2). Thus, the resulting siderophore encoded by the *irp* cluster of *V. ordalii* should be also piscibactin.

Like in *V. anguillarum*, the *irp* cluster genes of *V. ordalii* encode most functions needed for piscibactin synthesis and utilization, although an *entD* homologue is absent in this gene cluster when compared to the *P. damselae* subsp. *piscicida irp* cluster (Figure 1). The *entD* gene encodes a 4’-phosphopantetheinyl transferase that is required to activate the peptide synthesis domains of non-ribosomal peptide synthetases (NRPS) [41] and it is essential for piscibactin biosynthesis [23]. However, a homologue of this gene is present in the genome of *V. ordalii* as part of the *vab* gene cluster, encoding the siderophore vanchrobactin [4,26]. This *entD* homologue could provide in trans the function of a 4’-phosphopantetheinyl transferase necessary for piscibactin biosynthesis in *V. ordalii*. We have previously shown that although vanchrobactin could be synthetized by *V. ordalii*, it cannot be used as siderophore since the ABC transporters necessary for ferric vanchrobactin internalization are not present in the genome of *V. ordalii* [26].

### 3.2. Transcriptional Analysis and Iron Regulation of the Irp Gene Cluster of V. ordalii

**Figure 3:**
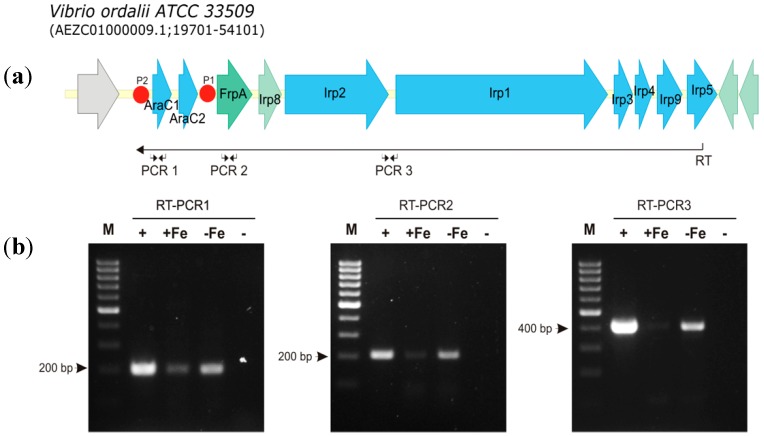
Transcriptional organization of the gene cluster putatively encoding biosynthesis and transport of siderophore piscibactin in *V. ordalii*. (**a**) The predicted gene functions are: biosynthesis, genes *irp1*, *irp2*, *irp3*, *irp4*, *irp5* and *irp9*; outer membrane receptor, *frpA*; transcriptional regulators, *araC1* and *araC2*; and inner membrane exporter of putative siderophore, *irp8*. Predicted promoters P1 and P2 containing Fur boxes are indicated by red dots. RT denotes the location of primer used in retrotranscriptase reaction while PCR 1, PCR 2 and PCR 3 indicate location of primers for detection of cDNA from piscibactin gene cluster. (**b**) results of three RT-PCR reactions designed to analyze the transcription of the *irp* gene cluster. Primer marked as RT, targeted to the 3′-end of *irp5* gene, was used to obtain a cDNA that spanned from *irp5* to *araC1*. This cDNA was then used as template for three PCR reactions targeted within *araC1* (RT-PCR1), *frpA* (RT-PCR2) and between *irp2* 3’-end and *irp1* 5’-end (RT-PCR3). M, size marker from 100 to 1000 bp. Negative controls (-) are RT-PCR reactions lacking reverse transcriptase. Positive controls (+) are PCR reactions using chromosomal DNA as template, +Fe: RT-PCR performed with cells grown under iron excess (TSB-1 + FeCl_3_ 20 µM); -Fe: RT-PCR performed with cells grown under iron limitation (TSB-1 + 2,2′-dipyridyl 60 µM).

To test if *irp* genes of *V. ordalii* were expressed, several RT-PCR (reverse-transcriptase PCR) reactions were performed. The results showed that the *irp* gene cluster is transcribed as a polycistronic mRNA that includes *araC1*, *araC2*, *frpA*, *irp1*-*5*, *irp8* and *irp9* genes (Figure 3). Therefore, all genes putatively encoding the synthesis, regulation and transport of piscibactin could be co-transcribed from the promoter P2 located upstream of *araC1* (Figure 3a). An identical result was found for the piscibatin *irp_ang_* cluster described in *V. anguillarum* RV22 [29]. This promoter contains a putative Fur box that would indicate that its activity is regulated by the transcriptional regulator Fur in an iron-dependent fashion [33]. An additional promoter P1, also containing a putative Fur box, was located upstream of *frpA* (Figure 3a). The *frpA* gene would encode the presumptive ferri-piscibactin outer membrane receptor while *araC1* would encode a putative AraC-type transcriptional regulator. Thereby, even though *irp* genes can be transcribed mainly from the promoter upstream of *araC1*, the existence of additional active promoters cannot be ruled out.

**Figure 4:**
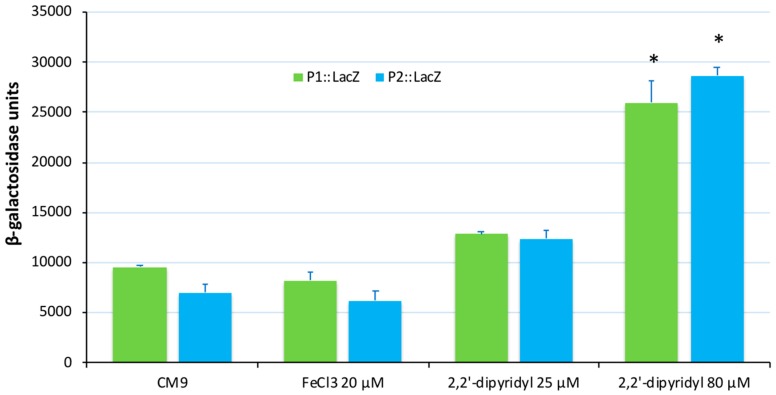
Transcriptional activity (β-galactosidase units) of *lacZ* fusions to P1 and P2 potential promoters of *V. ordalii*. β-galactosidase activities for promoter P1::*lacZ* and promoter P2::*lacZ* were measured in cells cultured in CM9 minimal medium, CM9 supplemented with 20 µM FeCl_3_, as an iron excess condition, and in two iron-limiting conditions: CM9 with 2,2′-dipyridyl 25 µM and CM9 with 2,2′-dipyridyl 80 µM. Three independent experiments were performed in triplicate. Bars represent average values with standard deviations indicated by error bars. The data were analyzed using ANOVA significance test (* *p* < 0.05).

In order to analyze the expression levels of the *irp* putative promoters P1 and P2, DNA fragments of ca. 700 nucleotides upstream of *frpA* and *araC1* genes (Figure 3a) were cloned into the plasmid pHRP309 upstream of a promoterless *lacZ* gene. Resulting plasmids were mobilized into *V. ordalii* Vo-LM-18 and the transcription levels of *lacZ* were measured by determining *β*-galactosidase activity under different conditions of iron availability (Figure 4). The use of the P*frpA* (P1) and P*araC1* (P2) presumptive promoters produced significant *β*-galactosidase activity when cells were cultured under a strong iron limitation (CM9 medium plus 2,2′-dipyridyl 80 µM). Under iron excess conditions (CM9 or CM9 plus FeCl_3_ 25 µM) the *β*-galactosidase activity of the P2 promoter was 75% of the P1 promoter (Figure 4), suggesting a higher basal activity for this promoter. However, under strong iron limitation, the P2 promoter seems to be 10% more active than P1, suggesting a tighter control by iron levels. These results demonstrate that the two promoter sequences could serve as transcriptional starts of the whole *irp* operon, and that both of them are strongly regulated by iron levels with slight variations between them.

### 3.3. Analysis of Iron-Regulated Outer Membrane Proteins

**Figure 5:**
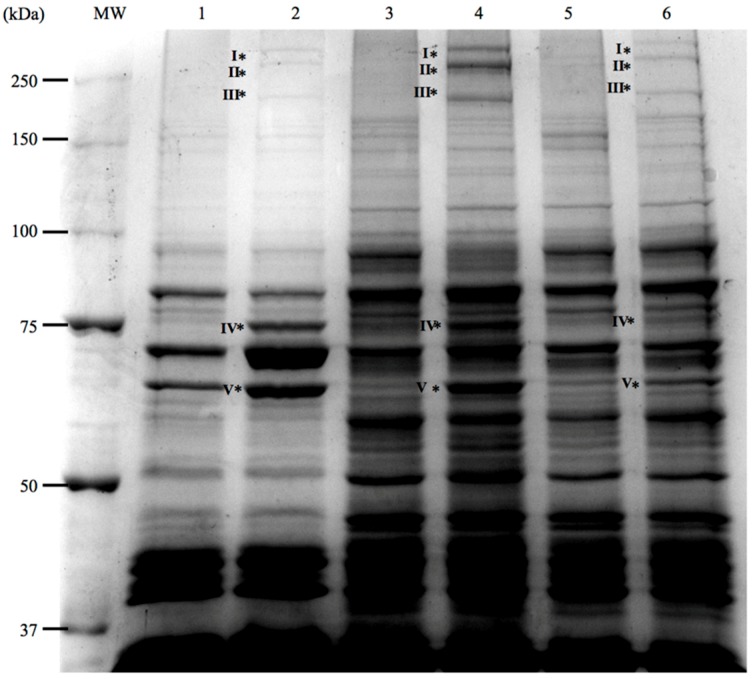
Representative SDS-PAGE gel showing outer membrane protein (OMP) profiles of *V. ordalii* strains under iron-rich and iron-limited conditions. MW: Molecular weight marker; 1: ATCC 33509^T^ under iron-excess conditions; 2: ATCC 33509^T^ under iron-limitation (TSB-1 + 2,2′-dipyridyl 45 µM); 3: Vo-LM-13 under iron-excess, 4: Vo-LM-13 under iron-limitation (TSB-1 + 2,2′-dipyridyl 90 µM); 5: Vo-LM-18 under iron-excess; and 6: Vo-LM-18 under iron-limitation (TSB-1 + 2,2′-dipyridyl 60 µM). *: Proteins expressed only under iron limitation and identified by PMF as follows: I, VabF (vanchrobactin synthesis); II, Irp1; III, Irp2 (piscibactin synthesis); IV, HuvS (heme receptor); V, FrpA (piscibactin receptor).

In Gram-negative bacteria some of the outer membrane proteins (OMP) are involved in iron uptake mechanisms, and most of them are regulated by iron. Thus, in order to detect the expression of OMPs involved in siderophore synthesis and transport in *V. ordalii* we investigated by SDS-PAGE the changes in the OMP profiles when cells were cultured under iron excess or under iron limitation. Some of these proteins could then be identified by PMF. As shown in Figure 5, clear changes in the OMP profile could be detected in three representative strains of *V. ordalii* when cells were cultured under iron deprivation (the strains were cultured in TSB-1 plus half the MIC of the iron chelator 2,2′-dipyridyl). Five main bands (Table 2) could be identified as proteins clearly regulated by iron since all them were present only in membrane fractions of cells grown under iron-limiting conditions. Three of these proteins were high-molecular weight proteins that were unequivocally identified by PMF as VabF (311 kDa band marked as I in Figure 5), Irp1 (270 kDa band marked as II in Figure 5) and Irp2 (224 kDa band marked as III in Figure 5). These three proteins correspond to NRPS enzymes involved in vanchrobactin (VabF) and piscibactin (Irp1 and Irp2) siderophore synthesis. Although NRPSs are cytosolic enzymes, it has been reported that some of them can form membrane-bound multi-enzymatic complexes, called siderosomes, on the inner leaflet of the cytoplasmic membrane [42,43], which could explain their detection in *V. ordalii* membrane fractions. Protein I showed 98% identity to VabF, a NRPS of *V. anguillarum* involved in vanchrobactin biosynthesis. Proteins II and III clearly correspond with Irp1 and Irp2, the two NRPS involved in the synthesis of piscibactin in *P. damselae* subsp. *piscicida* [23,27] (with similarities of 70% and 68%, respectively) and in *V. anguillarum* [29] (both proteins with similarities of 98%).

The other two differentially expressed bands with sizes of 79 kDa (band IV in Figure 5) and 71 kDa (band V in Figure 5) could be identified as the heme receptor HuvS, showing a 99% similarity to the homologous protein previously reported in *V. anguillarum* [44], and the piscibactin receptor FrpA, respectively. The later shows a 68% similarity with FrpA protein encoded by the plasmidic *irp* cluster of *P. damselae* subsp *piscicida* [27,40], and a 96% similarity with the FrpA protein reported in *V. anguillarum* [29].

From the analysis of the iron regulated OMP we could conclude that the *irp* gene cluster of *V. ordalii* must be fully functional, since biosynthetic and siderophore transport proteins are detected in cells grown under low iron conditions.

### 3.4. Identification of Siderophores in Cultures of V. ordalii

**Figure 6:**
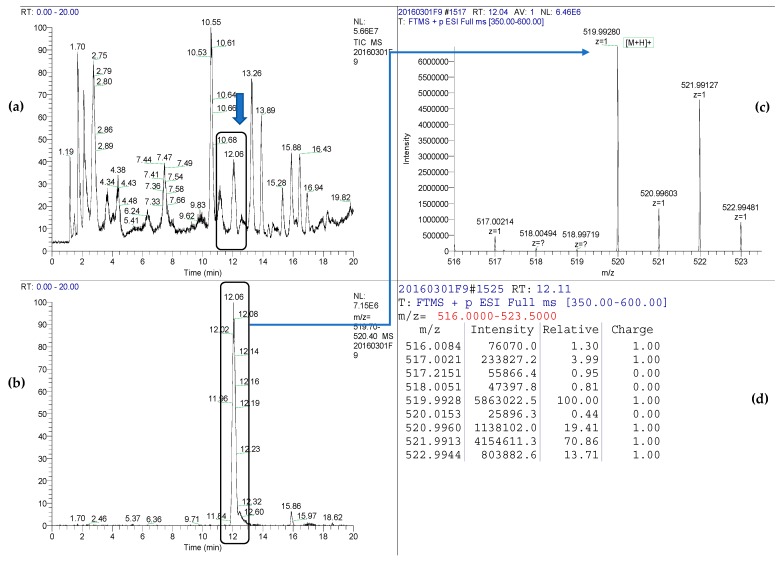
(**a**) LC-HRMS total ion chromatogram of the fraction containing piscibactin-Ga(III) complex eluted with H_2_O and CH_3_CN (1:1) from an OASIS^®^ HLB cartridge; (**b**) extracted ion chromatogram from *m*/*z* 519.7–520.4; (**c**) high resolution mass spectrum corresponding to the peak with *t*_R_ = 12.06 min; (**d**) isotopic ion abundance from *m*/*z* 516.0–523.5, including M+1/M (^13^C_1_/^12^C), calculated with Thermo Xcalibur software 3.0. LTQ-Orbitrap was operating at a resolving power of 30,000 (m/Δm) with a detection window setting around the compound of interest (between 350 and 600 dalton).

The genetic and bioinformatic analyses of the *irp* operon present in *V. ordalii*, as well as the identification of biosynthetic enzymes encoded by this cluster and induced under iron limitation, strongly indicate that *V. ordalii* would synthetize the siderophore piscibactin. In order to confirm the synthesis of this siderophore by *V. ordalii*, cell-free culture supernatants of strain Vo-LM-18 grown under iron-restricted conditions were examined for the presence of piscibactin as described in Material and Methods. The presence of the piscibactin-Ga(III) complex was confirmed on the basis of the accurate mass measurements and the characteristic isotopic cluster of the gallium complex (Figure 6). The ∆*m*/*z* results for monoisotopic and isotopic ions (M + 1) were below a tolerance acceptable value of 5 ppm and the δ RIA values were within the expected value for positive ion mode (16%) for a compound with a molecular mass range of 350–600 dalton measured in a LTQ-Orbitrap (Table 3) [45]. From these results, we can unequivocally conclude that piscibactin was present in the culture supernatants of *V. ordalii* Vo-LM-18.

Additionally, vanchrobactin was also detected in the cultures of strain Vo-LM-18 under iron restriction (data not shown). Solid Phase Extraction (SPE) using HLB cartridges of the cell-free culture supernatants of this strain followed by LC-MS analysis showed a peak with a retention time of 4.68 min, which displays a [M+H]^+^ ion at *m*/*z* 398.1676 (calculated for C_16_H_24_N_5_O_7_, *m*/*z* 398.1670) in its HRESIMS that corresponds to vanchrobactin [19,29].

## 4. Discussion

*V. ordalii* is the causative agent of vibriosis in several salmonid fish species farmed in several geographic areas around the world [1]. Although it was formerly classified as *V. anguillarum* biovar II, it was later recognized as a new *Vibrio* species [46]. Despite the similarities between both species, each of them causes quite different types of vibriosis [1,47] and some important genomic differences were reported between both species, for example the size of the genome of *V. ordalii* being 70% of that of *V. anguillarum* [4]. Both species also present important phenotypic differences [1]. Thus, the differences could also reach the virulence mechanisms used by each one to cause disease in fish. Among the variety of virulence factors present in *V. anguillarum*, the iron uptake systems are among the best studied [1,48]. However, these mechanisms are yet poorly known in *V. ordalii*. In a previous work we could detect the production of siderophores and suggested that piscibactin could be a siderophore being produced by this bacterium [26]. In the present research we could demonstrate that piscibactin is really the siderophore synthesized by *V. ordalii* under iron deprivation.

Vanchrobactin is a chromosomally encoded siderophore that is conserved among all *V. anguillarum* isolates as either environmental or pathogenic [21,49]. As noted above, all the genes necessary for vanchrobactin synthesis are also present in the genome of *V. ordalii* [4] and VabF, the NRPS that ensembles vanchrobactin, can be actually detected under low iron conditions (Figure 5). Moreover, we could detect the presence of vanchrobactin in the supernatants of *V. ordalii* cultured under iron limitation (data not shown). However, part of the required transporters, specifically the ABC transporters *fvtB-fvtE*, seem to be missed from the genome of *V. ordalii* [4]. Furthermore, although *fvtA*, the gene encoding the vanchrobactin outer membrane receptor of *V. anguillarum* [49], is present in the genome of *V. ordalii*, we could never detect any homolog to FvtA in the *V. ordalii* OMP profiles, suggesting that *fvtA* is not expressed. Thus, although *V. ordalii* also produces vanchrobactin, our results suggest that this bacterium is unable to use it as siderophore, confirming previously reported genomic and biological studies [4,26].

In addition to vanchrobactin, some strains of *V. anguillarum* lacking pJM1-type plasmids (that encode the synthesis of anguibactin [50]) produce also piscibactin as siderophore. The synthesis of piscibactin in *V. anguillarum* is favored at low temperatures since the transcriptional activity of the biosynthetic genes is three-times higher at 18 °C than at 25 °C [29]. Although in *V. anguillarum* vanchrobactin and piscibactin are simultaneously produced, the latter is a key virulence factor to infect fish whereas vanchrobactin seems to have a secondary role in virulence. This is in agreement with the observation that piscibactin seems to be the only siderophore used for iron uptake by *V. ordalii*. The fact that piscibactin synthesis in *V. anguillarum* is preferentially expressed below 18 °C also agrees with the usually lower optimal growth temperature of *V. ordalii* compared to *V. anguillarum* [2,46,51]. Thus, synthesis of piscibactin could be an adaptation to infect hosts that grow at low temperatures, and in these conditions piscibactin could be an efficient siderophore. It is noteworthy that piscibactin is the siderophore present in more species within the Vibrionaceae family than any other siderophore system. This wide distribution of piscibactin could be explained by a horizontal gene transfer (HGT) event that was followed by the action of diverse evolutionary forces [28]. As demonstrated in *P. damselae* subsp. *piscicida*, piscibactin is encoded by a pathogenicity island which resembles the high pathogenicity island (HPI) encoding the siderophore yersiniabactin in *Yersinia* [27,40]. The plasmid harboring this pathogenicity island could be transferred to other marine bacteria [27]. Acquisition of piscibactin genes by HGT could lead to the inactivation of other siderophore systems present in the ancient *Vibrio* genome. A similar event was demonstrated in *V. anguillarum*, in which the acquisition of the pJM1 plasmid, encoding the anguibactin siderophore system, led to the inactivation of vanchrobactin synthesis by a transposon harbored by the plasmid [21,52]. It is likely that the siderophore with the highest affinity for iron could have a selective advantage.

*V. ordalii* contains a significantly smaller genome than *V. anguillarum*, which explains the physical and ecological differences existing between both species [4]. Besides, this reduced genome suggests that *V. ordalii* may be immersed in the process of evolution toward an endosymbiotic lifestyle [5]. In this scenario, it is likely that vanchrobactin synthesis does not have any advantage, since its production could be more related to persistence into a marine environment than to pathogenesis [21,29]. Since piscibactin is a key virulence factor for *V. anguillarum* strains lacking the anguibactin system, and due to the close genetic relationship between both species, it is reasonable to speculate that the same will be true for *V. ordalii*. Multiple attempts to generate *V. ordalii* knock-out mutants (by the allelic exchange method previously used for *V. anguillarum* [29]) defective in piscibactin production were unsuccessful (data not shown). Further research is needed to try to generate piscibactin-deficient mutants in this bacterium to clearly demonstrate the involvement of piscibactin in the pathogenesis of vibriosis caused by *V. ordalii*.

In conclusion, *V. ordalii* produces piscibactin and vanchrobactin as siderophores when it is cultured under low iron conditions, but only piscibactin is used for iron uptake. The fact that piscibactin is a key virulence factor in other fish pathogens like *P. damselae* subsp. *piscicida* and *V. anguillarum*, highlights the importance of this siderophore in the pathogenesis of diseases caused by Vibrionaceae members in poikilothermic animals.

## Figures and Tables

**Table 1:** Primers used in this work.

Primers	Sequence (5′-3′) *	Amplified Fragment (bp)
**Amplification of potential promoters**		
**P1**		
Promoter 1_F	GCGTCTAGACACTTTGCCACCCACCATTA	879
Promoter 1_R	GCGGGATCCACGAATCGTCGTGTTGGCAT	
**P2**		
Promoter 2_F	GCGTCTAGACCGCTTAGAGAAACCAACGT	1165
Promoter 2_R	GCGGGATCCACGTTTCGGTAAGCGTATGG	
**Transcriptional regulation of *irp* gene cluster**		
RT	TTTGGAGATGAGTGCGACAC	
**PCR1**		
ARC1ordalii_F	GATATGCGCTTTGACTGCCA	196
ARC1ordalii_R	CTGTGAGACGGCATACAAGC	
**PCR2**		
FrpA_ordalii_F	CGGTGGTAATGCTCAAGGTG	204
FrpA_ordalii_R	TGGCTCGGTAGGTGTTCAAT	
**PCR3**		
Irp2_ordalii_F	AGCAGGCAACAAAGAGTGAG	413
Irp1_ordalii_R	GGGCGAATAACCAAACAAGC	

* Recognition sequences for restriction enzymes are underlined.

**Table 2:** Identification by peptide mass fingerprinting (PMF) of five proteins differentially expressed under iron limitation in SDS-PAGE gel showed in Figure 5.

Band in Gel (Figure 5)	Estimated Size (kDa)	Closest Homologues	Accession No.	Similarity (%)
Band I	311	VabF, *V. anguillarum*	CAJ45639.1	98
Band II	270	Irp1, *V. anguillarum*	WP_019281879.1	98
		Irp1, *P. damselae* subsp. *piscicida*	AKQ52532.1	70
Band III	224	Irp2, *V. anguillarum*	WP_019281878.1	98
		Irp2, *P. damselae* subsp. *piscicida*	AKQ52531.1	68
Band IV	79	HuvS, *V. anguillarum*	CAJ14788.1	99
Band V	71	FrpA, *V. anguillarum*	WP_019281876.1	96
		FrpA, *P. damselae* subsp. *piscicida*	AKQ52529.1	68

**Table 3:** List of ions observed in fraction eluted with H_2_O and CH_3_CN (1:1) ^a^, using LC-ESI-LTQ-Orbitrap in Positive-ion Mode ^b^.

Ion	Retention Time (min)	Detected [M+H]^+^	∆ m/z (ppm)	Ion Formula	Mean Intensity	δRIA (%)
Piscibactin-Ga(III)	12.08	519.99280	3.1	^12^C_19_H_21_^69^GaN_3_O_4_S_3_^+^	5.8 × 10^6^	-
		520.99603	3.4	^13^C_1_^12^C_18_H_21_^69^GaN_3_O_4_S_3_^+^	1.1 × 10^6^	−5.3
		521.99127	4.4	^12^C_19_H_21_^71^GaN_3_O_4_S_3_^+^	4.6 × 10^6^	-
		522.99481	4.1	^13^C_1_^12^C_18_H_21_^71^GaN_3_O_4_S_3_^+^	8.0 × 10^5^	5.9

^a^ Fraction mass 16.9 mg. ^b^ Fraction L3 was mixed with 3 µL of a 12 mg/mL solution of GaBr_3_/H_2_O just before injection.
